# DEMA: a distance-bounded energy-field minimization algorithm to model and layout biomolecular networks with quantitative features

**DOI:** 10.1093/bioinformatics/btac261

**Published:** 2022-06-27

**Authors:** Zhenyu Weng, Zongliang Yue, Yuesheng Zhu, Jake Yue Chen

**Affiliations:** Communication and Information Security Lab, Institute of Big Data Technologies, Shenzhen Graduate School, Peking University, Shenzhen 518055, China; Informatics Institute, School of Medicine, University of Alabama at Birmingham, Birmingham, AL 35294, USA; Communication and Information Security Lab, Institute of Big Data Technologies, Shenzhen Graduate School, Peking University, Shenzhen 518055, China; Informatics Institute, School of Medicine, University of Alabama at Birmingham, Birmingham, AL 35294, USA

## Abstract

**Summary:**

In biology, graph layout algorithms can reveal comprehensive biological contexts by visually positioning graph nodes in their relevant neighborhoods. A layout software algorithm/engine commonly takes a set of nodes and edges and produces layout coordinates of nodes according to edge constraints. However, current layout engines normally do not consider node, edge or node-set properties during layout and only curate these properties after the layout is created. Here, we propose a new layout algorithm, distance-bounded energy-field minimization algorithm (DEMA), to natively consider various biological factors, i.e., the strength of gene-to-gene association, the gene’s relative contribution weight and the functional groups of genes, to enhance the interpretation of complex network graphs. In DEMA, we introduce a parameterized energy model where nodes are repelled by the network topology and attracted by a few biological factors, i.e., interaction coefficient, effect coefficient and fold change of gene expression. We generalize these factors as gene weights, protein–protein interaction weights, gene-to-gene correlations and the gene set annotations—four parameterized functional properties used in DEMA. Moreover, DEMA considers further attraction/repulsion/grouping coefficient to enable different preferences in generating network views. Applying DEMA, we performed two case studies using genetic data in autism spectrum disorder and Alzheimer’s disease, respectively, for gene candidate discovery. Furthermore, we implement our algorithm as a plugin to Cytoscape, an open-source software platform for visualizing networks; hence, it is convenient. Our software and demo can be freely accessed at http://discovery.informatics.uab.edu/dema.

**Supplementary information:**

Supplementary data are available at *Bioinformatics* online.

## 1 Introduction

Network analysis provides a fundamental way to understand the biomolecular mechanisms systematically and the dynamics of signaling events (Koutrouli *et al.*, 2020; Ma'ayan, 2011). When dealing with a huge, complex biological network, layout algorithms play a vital role in revealing the underlying structure and sub-structure or functional associations so that the critical components or modules can be captured easily. Ideal network layouts are expected to not only present a global view of items with more space-filling and fewer overlapping nodes but also highlight specific parts of the views coupling with biological annotations (Kobourov, 2012; Pinto *et al.*, 2010; You *et al.*, 2010). Therefore, the mined critical biomarkers and models can further assist disease diagnosis (Bock and Ortea, 2020; Drier *et al.*, 2013; Liu *et al.*, 2016; Livshits *et al.*, 2015; Pian *et al.*, 2021; Zhang and Chen, 2010), cancer subtyping (Lafferty *et al.*, 2021; Mallavarapu *et al.*, 2020; Zhang and Chen, 2013) and personalized medicine (Chen *et al.*, 2007; Hamburg and Collins, 2010; Raghavan *et al.*, 2017). Various analytical strategies have been applied to biological network analysis, such as sub-network analysis in MCODE (Bader and Hogue, 2003), hub gene identification and geneset enrichment analysis, to extract critical genomic information (Khan *et al.*, 2020). Many layout algorithms incorporate biological factors, such as hubs (highly connected nodes) (He and Zhang, 2006), into a weighted network to generate layouts. Compared with the traditional Fruchterman and Reingold (FR) layout model (Fruchterman and Reingold, 1991), the recently proposed Force-Directed (FD) algorithm, Organic Layout (OL) algorithm (Cline *et al.*, 2007), BioLayout (aka. edge-weighted FD layout) (Enright and Ouzounis, 2001) and Forcealtas2 (FA2) (Jacomy *et al.*, 2014) show a protein–protein interaction (PPI) network’s hubs and overall network modularity clearly. To evaluate the PPI’s strength in a PPI network, different statistical methods were developed to capture the PPI score (Chen *et al.*, 2017; Langfelder and Horvath, 2008; Schaefer *et al.*, 2012). PPI score is incorporated by edge-weighted FR (EWFR) provided by Cytoscape (Shannon *et al.*, 2003) to generate a layout where the length of the edges reflects the degree of the relation. In addition, since the gene-set enrichment analysis (Yue *et al.*, 2015) has become popular, Enrichment Map (EMap), a layout plugin for Cytoscape, is developed to present relationships among gene sets (Merico *et al.*, 2010). In EMap, nodes represent gene sets and edges represent the ‘gene overlap’ between gene sets measured by the overlap ‘score’. By default, the overlap ‘score’ combined an equal-weighted Jaccard coefficient (50%) and overlap coefficient (50%), and the cutoff the overlap ‘score’ is 0.375. Deep learning network provides layouts using extracted features or embeddings from networks (Grover and Leskovec, 2016; Muzio *et al.*, 2021; Perozzi *et al.*, 2014; Tang *et al.*, 2015). However, none of the current layout algorithms integrate different types of biological factors into the layout generation and synthetically present a global view of genomic information.

Based on the above considerations, we propose a new layout algorithm, the distance-bounded energy-field minimization algorithm (DEMA). DEMA aims to incorporate biological network properties and prior knowledge information of the network to enhance network biology explorations and applications. It generates a layout where network hubs and the functional modules can be shown visually with controlled parameters including hub shrinking (HS) and the space filling (SF). A DEMA-optimized network layout may help users identify enriched biological pathways and enriched gene sets in a network context. In addition, the DEMA network layout may reveal gene signature patterns derived from functional genomics analysis using network visualization tools such as GeneTerrain (You *et al.*, 2010). The essential input of DEMA is a PPI network that describes the physical or co-expression relationships between genes. The biological factors analyzed by computational measures are categorized and defined in our layout. Interaction coefficient (IC) is defined as a measure to evaluate the reliability of the edges in the PPI network using statistical methods (Chen *et al.*, 2017). The IC can be extended to any biological feature that reflects the edge strength. Effect coefficient (EC) is defined as a measure to evaluate the correlation between genes based on gene expressions, such as the Pearson correlation coefficient (Langfelder and Horvath, 2008). EC can be substituted for any metric representing any correlation between the nodes in networks. Fold change (FC) is defined as the fold change of the gene expressions from case samples to control samples (Smyth, 2005). FC can be extended to any read-out values from experiments. DEMA can take one or more of IC, EC and FC as inputs and produce a layout by using the length of the edge to present these biological factors. Furthermore, the layout also considers gene-set annotation. By denoting which genes share similar functions, DEMA can group these genes together, and it can be extended to any grouping annotation of nodes.

The layout generated by DEMA has four properties:

1. *Topological property*: The layout generated by DEMA shows some modularities so that the hubs in the network can be observed. The layout generated by our method should make sure that the nodes do not overlap each other and can be seen clearly.
2. *Edge-weighted property*: If the edge that connects two nodes has a strong relation (EC or IC), then these two nodes will become close; otherwise, they are far away from each other.
3. *Node-weighted property*: Two nodes that share an edge will become close if they both have high node weights (FC) and have a positive association; otherwise, they are far away from each other.
4. *Grouping ability* **:** Gene-set annotation analysis is a popular technology to functionally analyze the gene sets in the network. Gene sets are defined as clusters of genes organized by different functionalities. The layout generated by DEMA can present the gene-set relation by grouping the genes that share similar functions.

Table 1 shows the comparison between DEMA and popular layouts provided by Cytoscape (Shannon *et al.*, 2003) and Gephi (Bastian *et al.*, 2009) software. DEMA is the only one layout algorithm that includes four kinds of properties.

**Table 1.** The comparison of different layout algorithms

Layout	Topological property	Node-weighted property	Edge-weighted property	Gene-set grouping property
OL (Cytoscape)	✓	✗	✗	✗
FA2 (Gephi)	✓	✗	✗	✗
FR (Gephi)	✓	✗	✗	✗
FD (Cytoscape)	✓	✗	✗	✗
EWFR (Cytoscape)	✗	✗	✓	✗
EMap (Cytoscape)	✗	✗	✗	✓
DEMA	✓	✓	✓	✓

## 2 Materials and methods

### 2.1 Model definition

Energy function-based layout methods are the commonly used methods for designing a layout. They model a graph layout as a physical system where nodes are attracted and repelled by different kinds of forces. We introduce a parameterized energy model where nodes are repelled by the network topology and attracted by a few biological factors, i.e., interaction coefficient (IC), effect coefficient (EC) and fold change (FC) of gene expression. The model is also suitable for a binary network where FD algorithms are usually applied. In the following sections, we discuss the base model, parameterized energy model and parameterized energy model with grouping. The base model corresponds to the DEMA layout with basic topological property (*L*_0_). Parameterized energy model corresponds to the DEMA layout with additional edge-weighted property (*L_e_*) and additional node-weighted property (*L_n_*). Parameterized energy model with grouping corresponds to the DEMA layout with additional gene-set grouping ability (*L_s_*).

### 2.2 Parameterized energy model

The energy function is composed of repulsion energy and attraction energy, which is defined as follows:

(1) $$E=K_{a}E_{a}+K_{b}E_{b}\;=\sum_{i}\sum_{j}\frac{K_{a}*R_{i}*R_{j}}{d_{i,j}}+\sum_{\;i,j\in \mathrm{NET}}\left(\frac{K_{b}*{FC}_{i}*{FC}_{j}}{w_{i,j}{-d}_{i,j}}-\frac{K_{b}*{FC}_{i}*{FC}_{j}}{w_{i,j}{-D}_{i,j}}\right),$$

where ${0<D}_{i,j}\leq d_{i,j}<w_{i,j}\;$and $D_{i,j}$ is an extremely small constant ($10^{-8}$ in default) that makes sure any two nodes will not collapse. *E_a_* denotes the repulsion energy and *E_b_* denotes the attraction energy. In the above equation, *NET* denotes the PPI network, *K_a_*, *K_b_* are control coefficients. When *K_b_*/*K_a_* is large, the attraction influence is strong to pull the nodes together, while the nodes are pushed away when *K_b_*/*K_a_* is small. *R_i_* is the RP score of gene *i*, *FC_i_* is the fold change (FC) of gene *i*, *d_i, j_* is the distance between gene *i* and gene *j*, and *w_i, j_* is a constraint weight to make the distance *d_i, j_* is smaller than *w_i, j_*.

Specifically, RP score *R_i_* proposed in Chen *et al.* (2006) separates the important nodes. It is defined as follows:

(2) $$R_{i}=e^{k*ln(\sum_{j,\mathrm{where}\left(i,j\right)\in \mathrm{NET}}{IC}_{i,j})-ln\sum_{j,\mathrm{where}\left(i,j\right)\in \mathrm{NET}}1},$$

where *IC_i,j_* is the interaction coefficient (IC) between gene *i* and gene *j*. *k* is set to two by default. Here, the PPI score between gene *i* and gene *j* is used as an interaction coefficient. *FC_i_* is the fold change (FC) of gene *i* expression values between the control samples and the case samples.

Constraint weight *w_i, j_* is defined as

(3) $$w_{i,j}=\max\left(1/\left(-\log\left(\frac{1-{IC}_{i,j}}{1+{IC}_{i,j}}\right)-\log\left(\frac{1-{EC}_{i,j}}{1+{EC}_{i,j}}\right)+K_{i,j}\right),\;D_{i,j}+\varepsilon \right),$$

where *K_i, j_* is set to the default of 1 and *EC_i, j_* is the effect coefficient (EC). Here, we use as EC the Pearson correlation of the genes. As the ranges of IC and EC are both [−1,1], the range of parameter w is (0, 1] due to the constraint of $D_{i,j}+\varepsilon$.

### 2.3 Base model

In some situations, we cannot get the FC, IC and EC. When this information is missing, the default values are set. For the attraction energy *E_b_*, *FC*, *IC* and *EC* are all set to be 1. Because $1/\left(-\log\left(\frac{1-{IC}_{i,j}}{1+{IC}_{i,j}}\right)-\log\left(\frac{1-{EC}_{i,j}}{1+{EC}_{i,j}}\right)+K_{i,j}\right)$ tends to 0 when the *IC* or *EC* tends to 1, $w_{i,j}=D_{i,j}+\varepsilon$.

In the repulsion energy $E_{a}$, due to *IC* equaling 1, the RP score is defined as

(4) $$R_{i}=e^{k*\ln(\sum_{j,\mathrm{where}\left(i,j\right)\in \mathrm{NET}}1)-ln\sum_{j,\mathrm{where}\left(i,j\right)\in \mathrm{NET}}1}.$$

It approximates the degree of the node. In the base model, the energy function is written as

(5) $$E=\sum_{i}\sum_{j}\frac{K_{a}*R_{i}*R_{j}}{d_{i,j}}+\sum_{i,j\in \mathrm{NET}}\left(\frac{K_{b}}{D_{i,j}+\epsilon {-d}_{i,j}}-\frac{K_{b}}{\epsilon }\right).$$

### 2.4 Parameterized energy model with grouping

Because gene-set analysis grows in popularity and increases the interpretability of data, we further improve our energy model by combining it with the gene set.

The gene-set energy is added into our model as the third item, which is defined as follows:

(6) $$E_{c}=\sum_{s\in \mathrm{gene}\;\mathrm{sets}}\sum_{i\in s}d_{i,c}^{2},$$

where *s* is a gene set, and *c* is the center position of *s*. Therefore, the energy function is

(7) $$E=K_{a}E_{a}+K_{b}E_{b}+K_{c}E_{c}.$$

### 2.5 Complete model of DEMA

The complete model of DEMA is

(8) $$E=K_{a}E_{a}+K_{b}E_{b}+K_{c}E_{c}=\sum_{i}\sum_{j}\frac{K_{a}*R_{i}*R_{j}}{d_{i,j}}+\sum_{\;i,j\in \mathrm{NET}}\left(\frac{K_{b}*{FC}_{i}*{FC}_{j}}{w_{i,j}{-d}_{i,j}}-\frac{K_{b}*{FC}_{i}*{FC}_{j}}{w_{i,j}{-D}_{i,j}}\right)+K_{c}\sum_{s\in \mathrm{gene}\;\mathrm{sets}}\sum_{i\in s}d_{i,c}^{2}.$$

### 2.6 Relation between parameter *w* and IC as well as EC

When IC or EC increases, the parameter *w* decreases (Supplementary Fig. S1), such that it can decrease the maximum distance between them. It is because of the constraint that the distance *d* between two genes should be smaller than their corresponding *w*. If a pair of genes have high IC and EC, which denote a strong relation, the pair should be close to each other.

### 2.7 Relation between the small energy system and parameter *w*

Assume that there are just two genes forming a small energy system composed of the repulsion energy and the attraction energy. If the other parameters in the energy system are fixed except for parameter *w*, when the distance is increasing, the repulsion energy decreases while the attraction energy increases. This results in an optimal distance between genes such that the total energy is minimized. Meanwhile, *w* will influence the optimal distance. When *w* is small, the optimal distance shrinks (Supplementary Fig. S2).

### 2.8 Relation between the group energy and distances from nodes to their center

For each group, when the distance between the node and the center increases, the group energy will increase. Minimizing the group energy pulls the nodes in the group close together and attracts each node close to the center of the group (Supplementary Fig. S3).

### 2.9 Finding a greedy solution

#### 2.9.1 Solution for parameterized energy model

Given the coordinates of a gene *i* in the 2D Euclidean plane by *p_i_* = (*x_i_, y_i_*), the distance *d_i, j_* between gene *i* and *j* is written as follows

(9) $$d_{i,j}=\sqrt{{\left(p_{i}-p_{j}\right)}^{2}}=\sqrt{{\left(x_{i}-x_{j}\right)}^{2}+{{(y}_{i}-y_{j})}^{2}}.$$

The goal of the algorithm is to find values for the coordinates of each node *i* to minimize the energy function *E*(*p_1_*, *p_2_*,…,*p_m_*). It is a constrained optimization problem; however, because the force is the derivative of potential energy (Kobourov, 2012), we can calculate the force exerted on one node and move it at a time to solve this problem. With respect to the position of one node *i*, we compute the gradient of the energy function and calculate the force exerted by other nodes.

For each gene *i*, the strength of the repulsion force exerted by node *j* is

(10) $$f_{i,j}^{a}=\frac{K_{a}*R_{i}*R_{j}}{d_{i,j}^{2}}.$$

And the direction is -(*p_j_*-*p_i_*). While the strength of the attraction force exerted by node *j* is

(11) $$f_{i,j}^{b}=\frac{K_{b}*{FC}_{i}*{FC}_{j}}{{\left(w_{i,j}-d_{i,j}\right)}^{2}}.$$

And the direction is *p_j_*-*p_i_*. Therefore, the total force is

(12) $$f=-\sum_{i}\sum_{j}f_{i,j}^{a}\left(p_{j}-p_{i}\right)+\sum_{\left(i,j\right)\in \mathrm{NET}}f_{i,j}^{b}\left(p_{j}-p_{i}\right).$$

After we get the direction of the node *i* moved by the force, the step length is to reduce the total energy. The step length is chosen to be 0.001 from a set L ranged from 0.001 to 1 in the greedy algorithm, which means to choose the maximum element l∈L such that the constraint will not be violated and the total energy will decrease after moving the point i with such a step length. The greedy algorithm can be implemented by binary search, resulting in a fast algorithm to find the result with O(log |L|).

#### 2.9.2 Solution for parameterized energy model with grouping

To solve the above model, we use the *EM* (*expectation and maximization*) algorithm to find the local optimal solution iteratively.

*In the expectation stage*, we fix the positions of the genes and determine the position of the center of the gene set according to the mean value of the positions of the corresponding genes, which makes the energy of the gene-set minimal.

*In the maximization stage*, we also move each gene according to the force exerted on it. For each gene *i*, the strength of the gene-set force exerted by its gene set(s) is

(13) $$f_{i}^{c}=K_{c}\sum_{s\in S}2d_{i,c},$$

where *S* is a set composed of the gene set containing gene *i* and *c* is the center of the gene set.

And the direction is *p_c_* − *p_i_*. Therefore, the total force is

(14) $$f=-\sum_{i}\sum_{j}f_{i,j}^{a}\left(p_{j}-p_{i}\right)+\sum_{\left(i,j\right)\in \mathrm{NET}}f_{i,j}^{b}\left(p_{j}-p_{i}\right)+\sum_{i}f_{i}^{c}\left(p_{c}-p_{i}\right)$$

After we get the direction of the point *i* to move on by the force, the step length is set to reduce the total energy. The step length is chosen to be 0.001 from a set L range from 0.001 to 1 in the greedy algorithm.

### 2.10 Heuristic start

To further converge the energy function to a minimum fast, a heuristic start is proposed. First, we construct the initial network by connecting each pair of nodes that share an edge.

Two kinds of energy compose the small energy system of each pair of nodes that share an edge in the formula (1).

From the derivation, we can find the optimal distance to minimize the energy system is

(15) $$d=\frac{w\sqrt{K_{a}*R_{i}*R_{j}}}{\sqrt{K_{b}*{FC}_{i}*{FC}_{j}+K_{a}*R_{i}*R_{j}}}$$

Then, we use the shortest path to connect all of the nodes to build a complete network.

After we define the distances between any pair of nodes, the formulation is transformed into the Kamada–Kawai (KK) model (Kamada and Kawai, 1989; Khoury *et al.*, 2012), and we can solve it according to the stress majorization algorithms (Gansner *et al.*, 2004; Khoury *et al.*, 2012). It is proved that it needs a few iterations to achieve an appealing layout by using the stress majorization. However, the layout cannot guarantee that the empirical distance between the nodes which share an edge is smaller than the edge weight since the empirical distance is sometimes larger than the ideal distance. To make the initial layout satisfy this constraint, we constantly replace *K_B_* by 2 * *K_B_* so that the optimal distance becomes smaller until the empirical distance between the nodes which share an edge is smaller than the edge weight. The layout generated by the heuristic start (HS) can be used as an initial layout for DEMA. Compared with random start (RS), DEMA with HS can show a layout with fewer iterations (Supplementary Fig. S4).

### 2.11 Network generation

The random networks are generated by NetworkX package (available from https://networkx.github.io/) in Python. They satisfy the scale-free property. The distribution of the degrees of the 100 nodes, 500 nodes and 1000 nodes are shown in Supplementary Figure S5, respectively.

## 3 Results

**Fig. 1.**
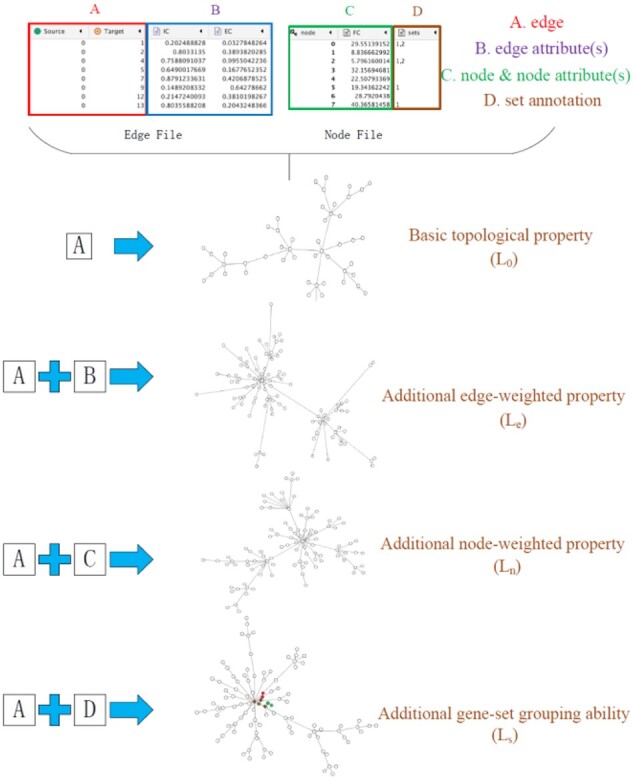
Illustration of DEMA. There are two files (node file and edge file) and four parts of them. Part A describes the PPI network. Part B describes the attributes of the edges. Part C describes the attributes of the nodes. Part D annotates the sets that the nodes belong to. Among them, Part A is the essential input and combines with other parts to perform additional corresponding functional properties

DEMA is a layout algorithm implemented as an easily operated Cytoscape plugin (download at http://discovery.informatics.uab.edu/dema). The input to DEMA consists of two files: the edge file that describes the attributes of the edges connecting the nodes and the node file that describes the properties of the nodes. As depicted in Figure 1, there are four properties of DEMA corresponding to four parts (A, B, C, D) of inputs, respectively. Part A is a PPI network and the necessary input to DEMA. Other parts are optional. The four properties will be introduced one by one. A case study is given at the end.

### 3.1 Topological property

As depicted in Figure 1, by importing the PPI network that uses edges to describe the network, DEMA can generate a layout with topological modularity (L_0_).

**Fig. 2.**
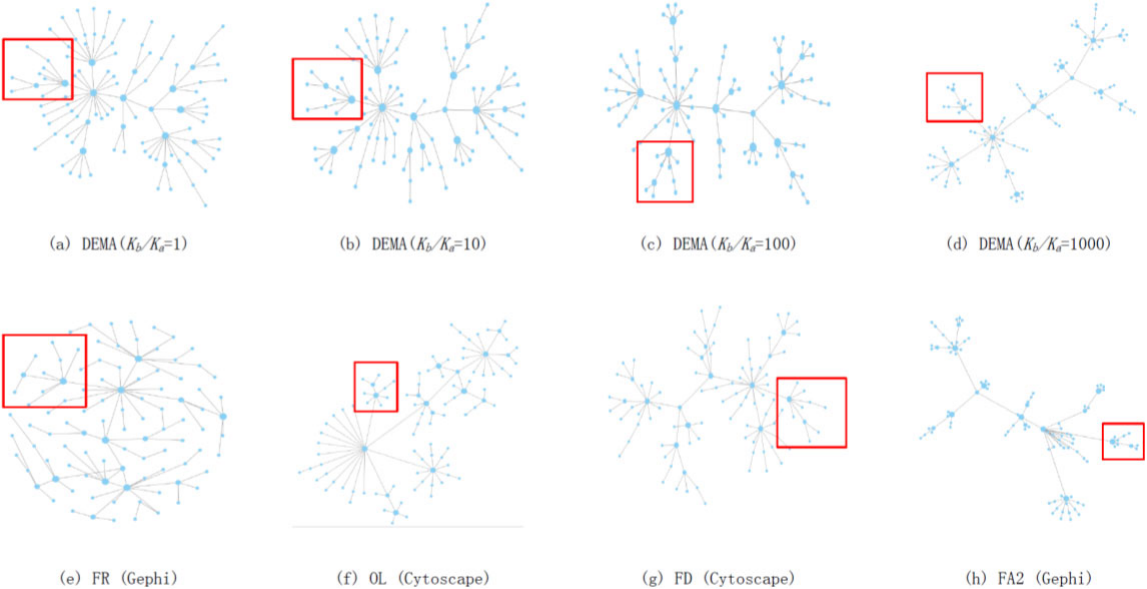
A comparison of different network layouts with network graph node size *n* = 100. (**a–d**) The layouts drawn by DEMA with different parameters K_b_/K_a_ are described. (**e–h**) The layouts drawn by different layout algorithms

The comparison between DEMA and other layouts with a random network of 100 nodes satisfying scale-free property (Barabási, 2009) is shown in Figure 2. Cytoscape and Gephi provided the best-performed layout with their default parameters that we adopted in the comparison. Figure 2(a–d) is generated by DEMA with different values of control the parameter *K_b_/K_a_*. Figure 2(e–h) are the popular layouts provided by software Cytoscape and Gephi. They are FR model (Fruchterman and Reingold, 1991), FD layout (http://www.prefuse.org), Organic layout (OL) (Cline *et al.*, 2007) and ForceAtlas2 (FA2) (Jacomy *et al.*, 2014), respectively. From Figure 2(a–d), we can see that when *K_b_/K_a_* becomes large, the modularity in the layout is more obvious. However, the distance between the nodes surrounding the hubs and the hubs becomes small, which makes it difficult to observe the surrounding nodes. Compared with other layouts, we can see that (e) subfigure roughly corresponds to (a), and the hubs in the layouts are both not obvious. (f) roughly corresponds to (b), while there is no hierarchy in the hub and its neighborhood in (f). In Figure 2b, we can see that the edge between the small hub and the big hub is longer than the edges between other nodes and the big hub in the crop regions in the red rectangle frames. In contrast, in Figure 2f, the edge between the small hub and the big hub is the same length as the edges between other nodes and the big hub. (g) roughly corresponds to (c), while some edges cross in (g). (h) roughly corresponds to (d). Both of them clearly show the hubs, but the nodes cannot be observed clearly.

**Fig. 3.**
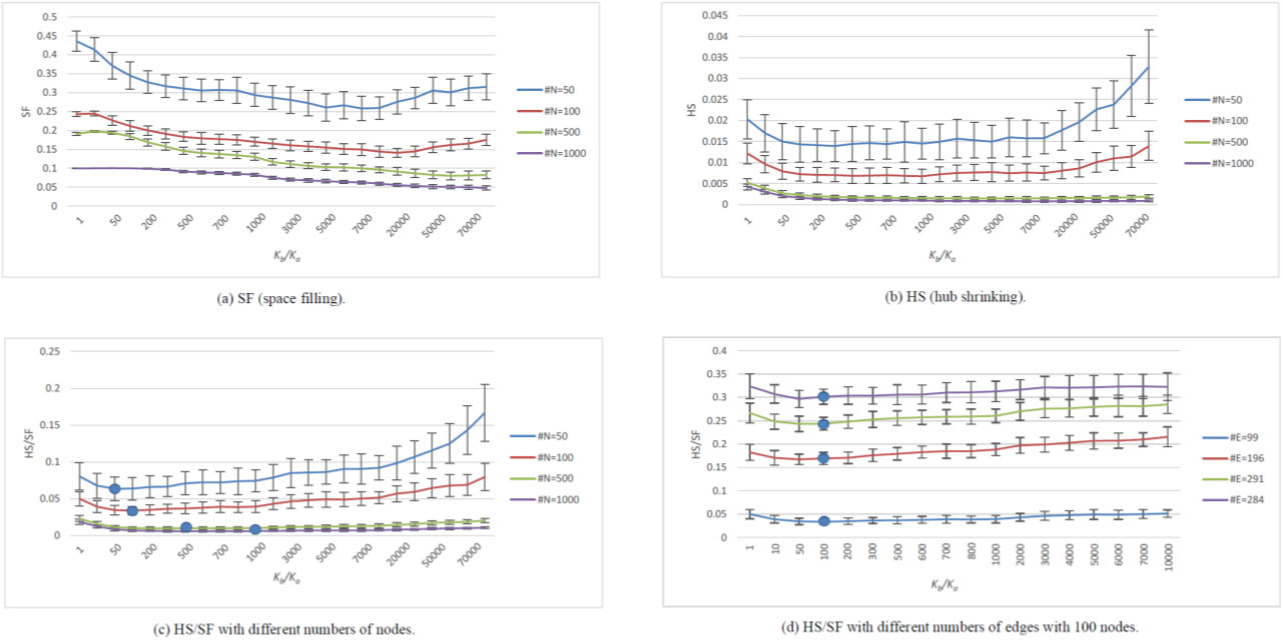
Effects of layout by varying network sizes and DEMA K_b_/K_a_ parameters. #N denotes the number of the nodes. #E denotes the number of the edges. (**a**) Space-filling (SF) is used to evaluate visual clarity. The larger, the better. (**b**) Hub shrinking (HS) is used to evaluate structural modularity. The smaller, the better. (**c**) and (**d**) use HS/SF to achieve a balance between the visual clarity and the structural modularity. The smaller, the better. The round dots in (c) indicate the optimal K_b_/K_a_ values with the lowest of HS/FS ratio. The round dots in (d) indicate the optimal choice for K_b_/K_a_ determined by the input node size

To choose a proper parameter *K_b_/K_a_* for DEMA, we access the network modularity by evaluating visual clarity and network modularity. With a proper parameter *K_b_*/*K_a_*, DEMA can clearly observe the hubs and the nodes. We use space filling (SF) to evaluate the visual clarity and hub shrinking (HS) to evaluate the structural modularity. First, we normalize the nodes’ coordinates at [0, 1] for each axis. Then, we build a grid and see what percentage of the small squares in the grid are occupied by the nodes; it is the SF measure. The larger the percentage, the more dispersed the nodes are. For the HS measure, the coordinates of the nodes are also normalized at first. We then calculate the interaction arithmetic mean of square distance between each pair of nodes that share an edge. Obviously, the smaller the HS value, the more the nodes shrink to the hubs. The networks with 50 nodes, 100 nodes, 500 nodes and 1000 nodes are randomly generated. Each kind of network is generated 100 times. SF and HS are calculated and shown in Figure 3a and b. For SF, the number of the small squares in a grid is almost twice the number of the nodes. Although there are some unstable situations, HS and SF generally decrease as *K_b_/K_a_* increases. SF stabilizes after it reaches a valley, while HS bounces up after *K_b_/K_a_* crosses a threshold. The reason that HS bounces up is that the distance between the hubs increases, and it affects HS more than the distance between the hub and the peripheral nodes does. To achieve a balance between SF and HS, we calculate HS/SF. The result is shown in Figure 3c. From the figure, we can see that the best parameter for the smallest HS/SF is usually close to the number of nodes. Also, from Figure 3d, we can observe that given 100 nodes with different numbers of edges, the best parameter for the smallest HS/SF is still close to the number of nodes. Therefore, the default value of *K_b_/K_a_* is the number of nodes.

The HS/SF values for the layouts shown in Figure 2 are calculated in Table 2. From the results, we can see that DEMA achieves the lowest value and produces the best balance between the hub shrinking (HS) and the space-filling (SF).

**Table 2.** HS/SF for different layouts

	FR	FD	OL	FA2	**DEMA (**K_b_/K_a_ **= 100)**
HS	0.026	0.010	0.015	0.008	0.008
SF	0.210	0.418	0.382	0.219	0.352
HS/SF	0.052	0.025	0.040	0.036	**0.023**

The bold value indicates the DEMA performed the best (the lowest value in the HS/SF metric).

### 3.2 Edge-weighted property

In Figure 1, Part B of the edge file includes the two computational biological factors, interaction coefficient and effect coefficient, as the attributes of the edges. Interaction coefficient (IC) is defined as a measure to evaluate the reliability of the edges in the PPI network using statistical methods [11]. Effect coefficient (EC) is defined as a measure to evaluate the correlation between genes based on gene expressions, such as the Pearson correlation coefficient [12]. By importing the PPI network and these biological factors represented as the edge weights, the layout in the figure not only shows the network modularity but also reflects the relationship between the nodes by the length of the edge (*L_e_*). If any two nodes that share an edge have a strong IC or EC, they are close to each other; otherwise, they are far away from each other.

We use the *P*-value to show that the length of the edge can demonstrate the degree of the relationship. For IC and EC, the edges are sorted according to their values from largest to smallest. We select as top edges the top *p* percent of the total edges and calculate the average length of the top edges. Then, we randomly select *p* percent of the edges and check whether the average length of the selected edges is smaller than the average length of the top edges. The selection is repeated 10 000 times. The *P*-value is calculated as the probability that the average length of the randomly selected edges is smaller than the average length of the top edges. We generate six kinds of random networks, and the IC and EC values of the networks are randomly generated 100 times. These six kinds of random networks are network 1 (100 nodes and 99 edges), network 2 (100 nodes and 196 edges), network 3 (100 nodes and 291 edges), network 4 (500 nodes and 499 edges), network 5 (500 nodes and 996 edges) and network 6 (500 nodes and 1491 edges). From Supplementary Figures S6 and S7, we see that the *P*-values of EC and IC are below 0.05 for the network with 100 nodes and 500 nodes. They are significant. It means that our parameterized layout not only has network modularity but also reflects the relationship between any two nodes that share an edge by the length of the edge.

### 3.3 Node-weighted property

In Figure 1, Part C of the node file includes fold change, a biological factor, as the attribute of the node. Fold change (FC) is defined as the fold change of the gene expressions from case samples to control samples (Smyth, 2005). By importing the PPI network and the FC of nodes, the layout in the figure not only shows the network modularity but also reflects the relation between the nodes by the length of the edge (*L_n_*). If any two nodes that share an edge both have the high FC, they are close to each other.

We also use the *P*-value to show that the length of the edge can demonstrate the degree of the relationship. If two nodes that share an edge both have a high FC value, they should be close to each other; otherwise, they are far away from each other. The value of the edge is defined as the product of the values of the two nodes that the edge connects. The edges are then sorted from largest to smallest. We select as top edges the top *p* percent of the total edges and calculate the average length of the top edges. Then, we randomly select the *p* percent of the edges and check whether the average length of the selected edges is smaller than the average length of the top edges. The selection is repeated 10 000 times. The *P*-value is calculated as the probability that the average length of the randomly selected edges is smaller than the average length of the top edges. We generate a random network, and the values of FC for the networks are randomly generated 100 times. From Supplementary Figure S8, we see that the *P*-values of FC are all below 0.08 no matter for the network with 100 nodes or 500 nodes. They are significant.

### 3.4 Gene-set grouping ability

**Fig. 4.**
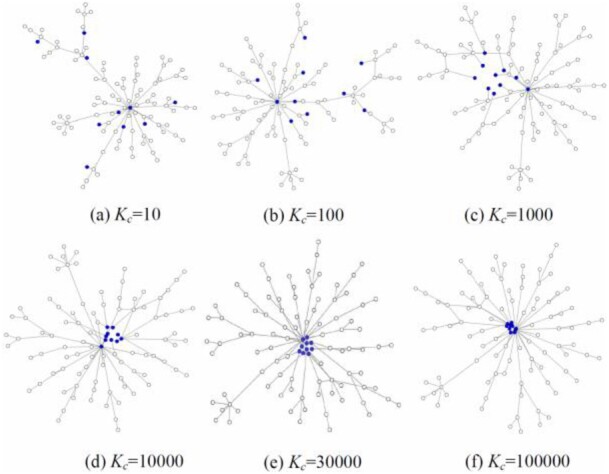
Effects of DEMA layout by varying *Kc*. The solid nodes represent the nodes in the set

In Figure 1, Part D of the node file includes gene-set annotation, denoting what gene sets each gene is from. By importing the PPI network and the gene-set annotation, the layout can show the additional grouping ability (*L_s_*). From the figure, one can see that in some rows of the set column, multiple sets are denoted and separated by commas. Some genes may belong to multiple sets. DEMA will group the genes in the same set. In DEMA, there is a parameter *K_c_* to control the degree to which the genes in the same set are grouped. As described in Figure 4, the genes in the set will become closer as *K_c_* increases.

**Fig. 5.**
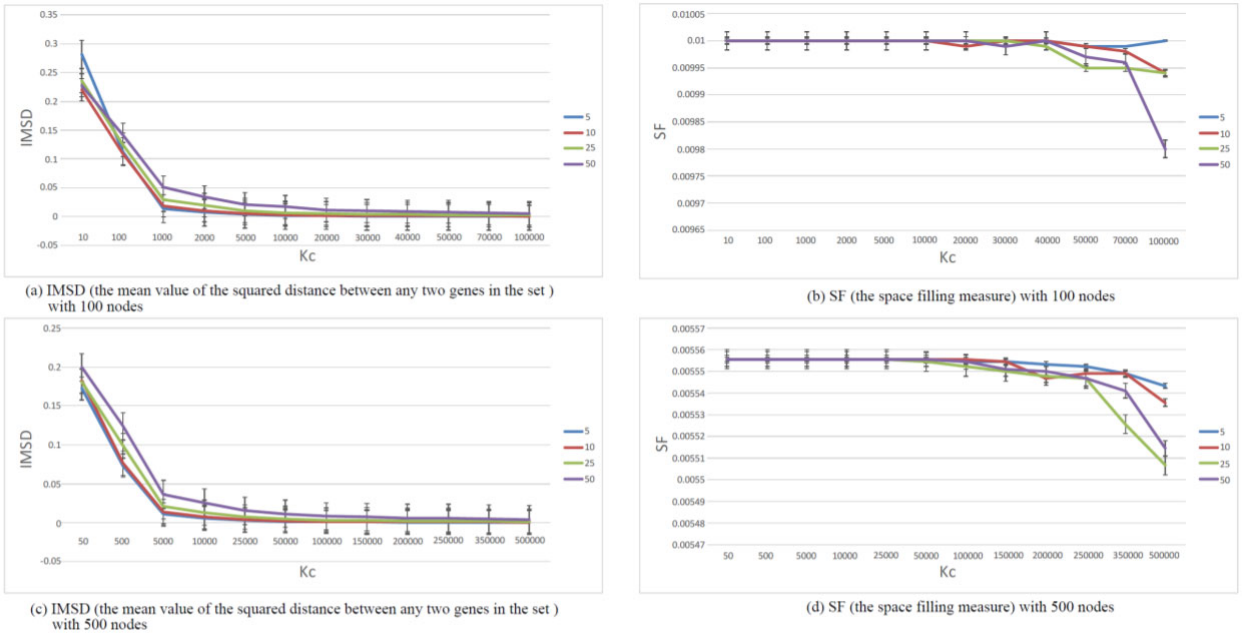
DEMA gene-set grouping ability with varying K_c_. In (**a**) and (**c**), (IMSD) is defined to measure the distance between the nodes in the set. In (**b**) and (**d**), SF is defined to measure the space filling for all the nodes. The legend represents the gene subsets (5, 10, 25 and 50%) randomly selected from all the genes, respectively

To evaluate the grouping performance, we define two measures, the inside mean value of the squared distance (IMSD) and space filling (SF). IMSD is defined as the mean value of the squared distance between any two genes in the set divided by the mean value of the pairwise squared distance among all the input genes. SF is defined in the above part, which is used to evaluate visual clarity. We generate 10 random networks with 100 nodes and calculate the IMSD and SF for four gene sets. They include gene subsets (5, 10, 25 and 50%) randomly selected from all the genes, respectively. The averaged results are shown in Figure 5a and b. From Figure 5a, we can see that IMSD becomes smaller as *K_c_* increases, which shows that the nodes in the group become closer. From Figure 5b, we can see that SF is almost the same before *K_c_* is equal to 40 000. When *K_c_* is larger than 40 000, SF declines rapidly, which means that some nodes overlap. We generate 10 random networks with 500 nodes and calculate the IMSD and SF for four gene sets. The averaged results are shown in Figure 5c and d. In Figure 5c, IMSD also becomes smaller as *K_c_* increases. And in Figure 5d, SF is almost the same before *K_c_* is equal to 150000. When *K_c_* is larger than 150 000, SF also declines rapidly. Therefore, the default *K_c_* value is defined as 300 times as the number of nodes to make a balance between the visual clarity and the grouping degree.

### 3.5 Case study of autism spectrum disorder

**Fig. 6.**
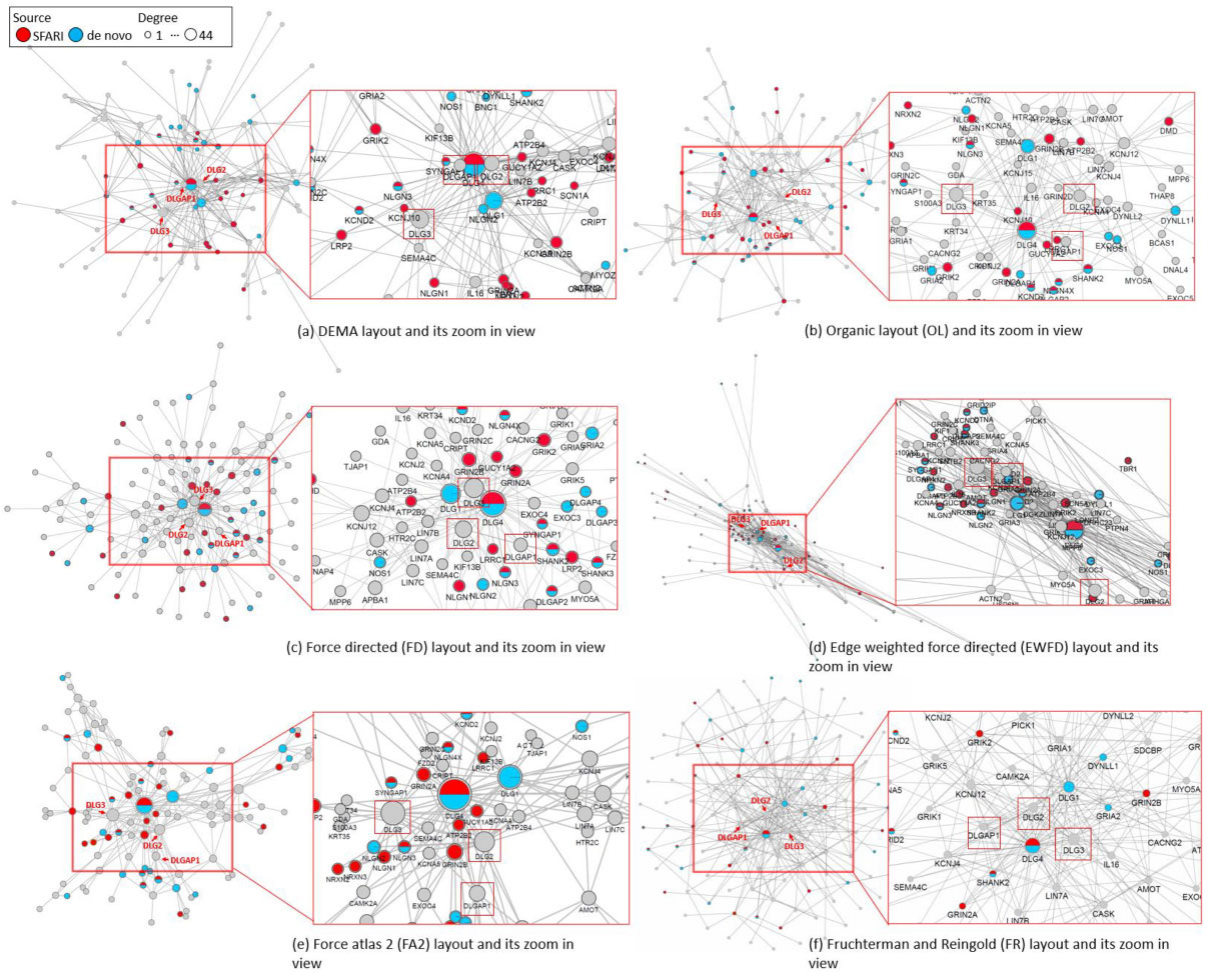
Biological interpretability for ASD (autism spectrum disorders). The blue nodes indicate the genes from *de novo* CNVs and the red nodes indicate the gene from SFARI reference. DLG2, DLG3 and DLGAP1 are highly connected to the ASD-related genes. (**a**) Organic layout, (**b**) DEMA layout, (**c**) Force-directed (FD) layout, (**d**) Edge-weighted force-directed (EWFD) layout, (**e**) Force atlas 2 (FA2) layout and (**f**) Frutchterman and Reingold (FR) layout (A color version of this figure appears in the online version of this article)

Autism spectrum disorder (ASD) is analyzed by DEMA as a case study. Autism is a complex disease, and it is still difficult to determine its etiology. In the paper Li *et al.* (2014), a human PPI network is built and decomposed into 817 topological modules based on the human protein interactome from BioGrid (Stark *et al.*, 2011) (Supplementary File S1). Among these modules, Module #13 shows significant enrichment related to ASD and includes 199 genes. In this module, the SFARI reference ASD genes and *de novo* CNVs are denoted (Supplementary File S1). We took the cluster #13’s genes and extracted the subset of the PPIs associated to those genes in the cluster #13 directly from the supplemental of the paper (Stark *et al.*, 2011). This module is used to draw a layout by DEMA, organic layout (OL), FD, edge-weighted force-directed (EWFD), Forcealtas2 (FA2) and Fruchterman and Reingold (FR) layout (Fig. 6), respectively. The nodes in red are SFARI reference genes, and the nodes in blue are *de novo* CNVs. The other layouts only show which genes are connected to the related genes. However, the layout by DEMA can group the related genes together and observe the hubs that connect to these related genes. DEMA performances the best in both SFARI and *de novo* gene groupings measured by IMSD scores (Supplementary Table S1). Three candidate genes are denoted by the arrows, which will be further analyzed in the following. One can see that DEMA can group the genes in the same set together. By zooming in DEMA, in Figure 6a, DLG2 (Jiangxie *et al.*, 2014), DLG3 (Kantojärvi *et al.*, 2011) and DLGAP1 (Egger *et al.*, 2014) are highly connected to the ASD-related genes, which are also related to ASD according to the literature. These genes are considered to be new candidate genes related to ASD.

### 3.6 Case study of Alzheimer’s disease

**Fig. 7.**
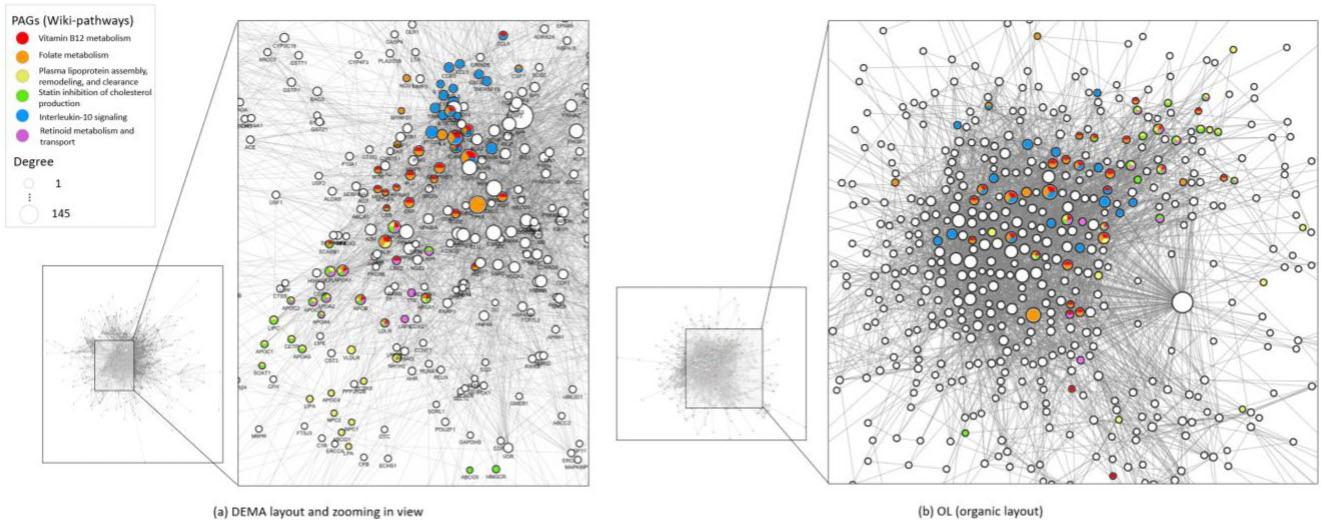
Biological interpretability of Alzheimer’s disease (AD). (**a**) DEMA layout with enriched PAG (wiki-pathway) annotations and zooming in view of DEMA layout. (**b**) Organic layout. The highlighted genes in different colors represent the members of PAGs

To illustrate the biologically functional gene groups in a heterogeneous disease like Alzheimer’s disease (AD), an important parameter *K_c_* in DEMA has been designed to group and visualize the acknowledged-based gene sets in disease gene networks. In our study, 680 candidate genes in view of genetic risk were collected from the AlzGene database (https://www.alzforum.org/alzgene). To construct the gene network, the PPI were retrieved from the HAPPI-2 database (Chen *et al.*, 2017) using the quality more than or equal to 4-star (PPI score ≥ 0.75), and the PPI scores were used as IC scores (Supplementary File S2). There were 558 genes connected by 3459 PPIs. With the aim of highlighting the important genes in the network. After proceeding with the gene enrichment analysis using PAGER 2.0 database (Yue *et al.*, 2015, 2018), we found 6 highly relevant pathways using the false discovery rate (FDR) ≤ 0.05, overlaps ≥ 15 and similarity score ≥ 0.1. The similarity scores were calculated using the methods in (Huang *et al.*, 2012) (Table 3). After setting the parameters *K_b_*/*K_a_* = 558 and *K_c_* = 150 000 suggested by the DEMA, the AD gene network was visualized as well as the crucial genes in pathways were grouped with clear functional patterns in Figure 7a compared to the organic layout in Figure 7b. Several key genes with high-genetic risk were revealed by overlapping four or five pathways in Figure 7, Supplementary Table S2 and File S2. For instance, APOA1, APOB, LDLR, APOE and ABCA1 involve in at least four pathways, ‘Vitamin B12 metabolism’, ‘Folate metabolism’, ‘Plasma lipoprotein assembly, remodeling, and clearance’, ‘Statin inhibition of cholesterol production’ and ‘Retinoid metabolism and transport’. In DEMA, these genes were grouped coherently. TNF, IL6, IL1B, CCL2 and ICAM1 are located near each other, and these genes are involved in the three pathways, ‘Vitamin B12 metabolism’, ‘Folate metabolism’ and ‘Interleukin-10 signaling’. ALB locates near APOE, and it shares three identical pathways with APOE. APOA2, APOC3, APOA4 and APOC2 locate near to each other. These genes share the three pathways ‘Plasma lipoprotein assembly, remodeling, and clearance’, ‘Statin inhibition of cholesterol production’ and ‘Retinoid metabolism and transport’. SCARB1 locates at the intersection of the pathway group, ‘Vitamin B12 metabolism’, ‘Folate metabolism’ and ‘Plasma lipoprotein assembly, remodeling, and clearance’. In all, DEMA has successfully highlighted and grouped the functional genes together with gene-set-based information, which possessed considerable application potential for further investigation.

**Table 3.** The information of enriched PAGs from wiki-pathway in Alzheimer’s disease

ID	Name	Size	Overlap	Similarity	Adj.P	Color
WAG002004	Vitamin B12 metabolism (WP1533)	53	29	0.142	3.84E−38	Red
WAG002021	Folate metabolism (WP176)	72	30	0.116	7.30E−35	Orange
WAG002786	Plasma lipoprotein assembly, remodeling and clearance (WP4129)	67	25	0.102	2.64E−28	Yellow
WAG002857	Statin inhibition of cholesterol production (WP430)	31	19	0.144	1.92E−26	Green
WAG002729	Interleukin-10 signaling (WP4063)	46	20	0.109	3.08E−24	Blue
WAG002606	Retinoid metabolism and transport	32	15	0.11	7.05E−19	Purple

*Note*: The ‘ID’ represents the PAG ID, in which the details of PAG can be retrieved using the url http://discovery.informatics.uab.edu/PAGER/index.php/geneset/view/[PAG_ID]. The ‘Overlap’ represents the number of overlapped genes between the queried gene list and the PAG gene members. The ‘similarity’ is calculated based on the combination of overlap coefficient and Jaccard index. The ‘Adj.P’ represents the adjusted *P*-values.

## 4 Conclusion and discussion

DEMA introduced a parameterized energy model to integrate the critical biological factors, the biological network analysis, the strength of PPIs, the gene-to-gene correlations, the gene strength and the functional gene groups. To find a local optimal solution iteratively, we applied EM (expectation and maximization) algorithm. Thus, DEMA delivers sub-optimal solutions to the global problem, and the local-field energy needs to be minimized using the stacked forces to continue the search beyond local optimality. To search for the shortest paths in the heuristic start, we applied the stress majorization algorithms, and it is faster than the random start to get the initial coordinates. Additionally, to evaluate the structure modality in comparing DEMA to the other layout algorithms, we introduced space filling (SF) and hub shrinking (HS). DEMA takes the lead in balancing between the HS and SF. We statically validated the node, edge, and grouping properties in the synthetic networks. DEMA can be extended the adaptivity in substituting the designed biological factors (IC, EC, FC and geneset grouping) to any equivalent feature in other Omics, such as metabolomics network, drug–drug interaction network and hybrid networks across different biological domains. Further, we performed two real-world case studies using biological networks. Since DEMA adapts the energy-based algorithm, the basic model using an unweighted network may produce a layout similar to the organic layout. However, given the biologically functional annotations, DEMA makes a big difference by revealing biological groupings and those surrounding candidates intuitively. We expect DEMA to be a major tool in network analytics.

## Supplementary Material

btac261_Supplementary_Data
