# Supplementary material for: DEMA: a distance-bounded energy-field minimization algorithm to model and layout biomolecular networks with quantitative features
Source: Bioinformatics. 2022 Jun 27;38(Suppl 1):i359–68. doi: 10.1093/bioinformatics/btac261 (PMC9235497; doi:10.1093/bioinformatics/btac261)
Supplement: btac261_Supplementary_Data [file btac261_supplementary_data.pdf]

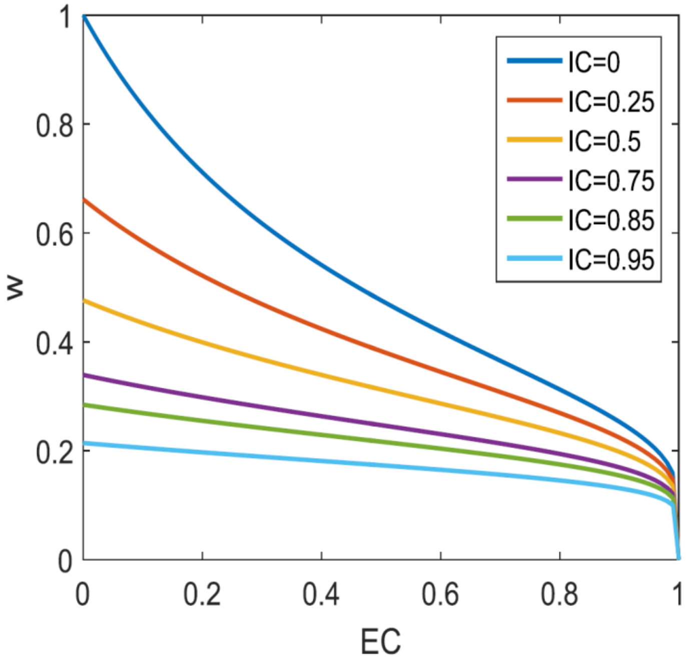

Fig_S1 Relationship between parameter w and IC as well as EC. When IC or EC increase, the parameter w decreases.

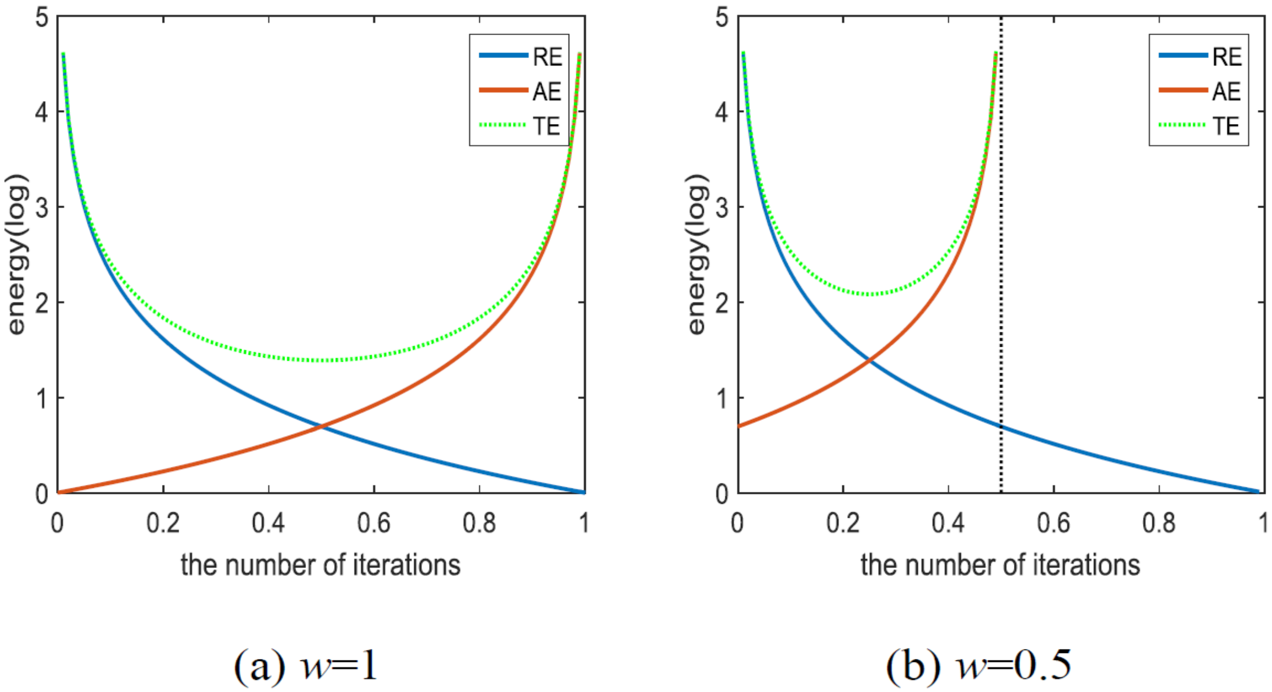

Fig_S2 Relationship between small energy system and parameter w. RE
denotes the repulsion energy. AE denotes the attraction energy. TE de-notes the total energy. When parameter w decreases, the optimal distance to achieve minimum total energy in the system decreases. When w = 1, the optimal distance is 0.5. When w = 0.5, the optimal distance is almost 0.24.

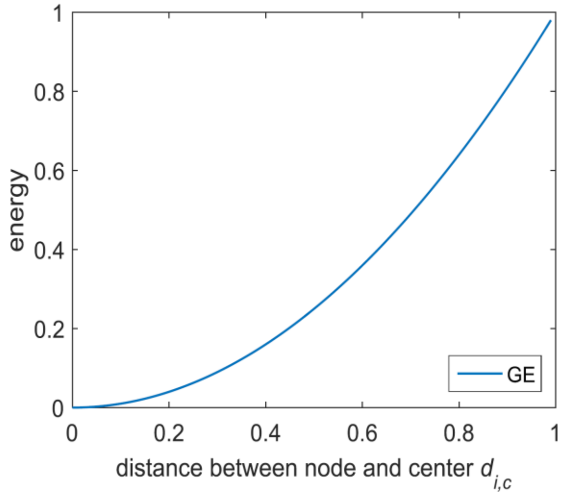

Fig_S3 Relationship between gene-set energy and distance from node to center. GE denotes the group energy. When the distance be-tween the node and the center increases, the group energy will increase.

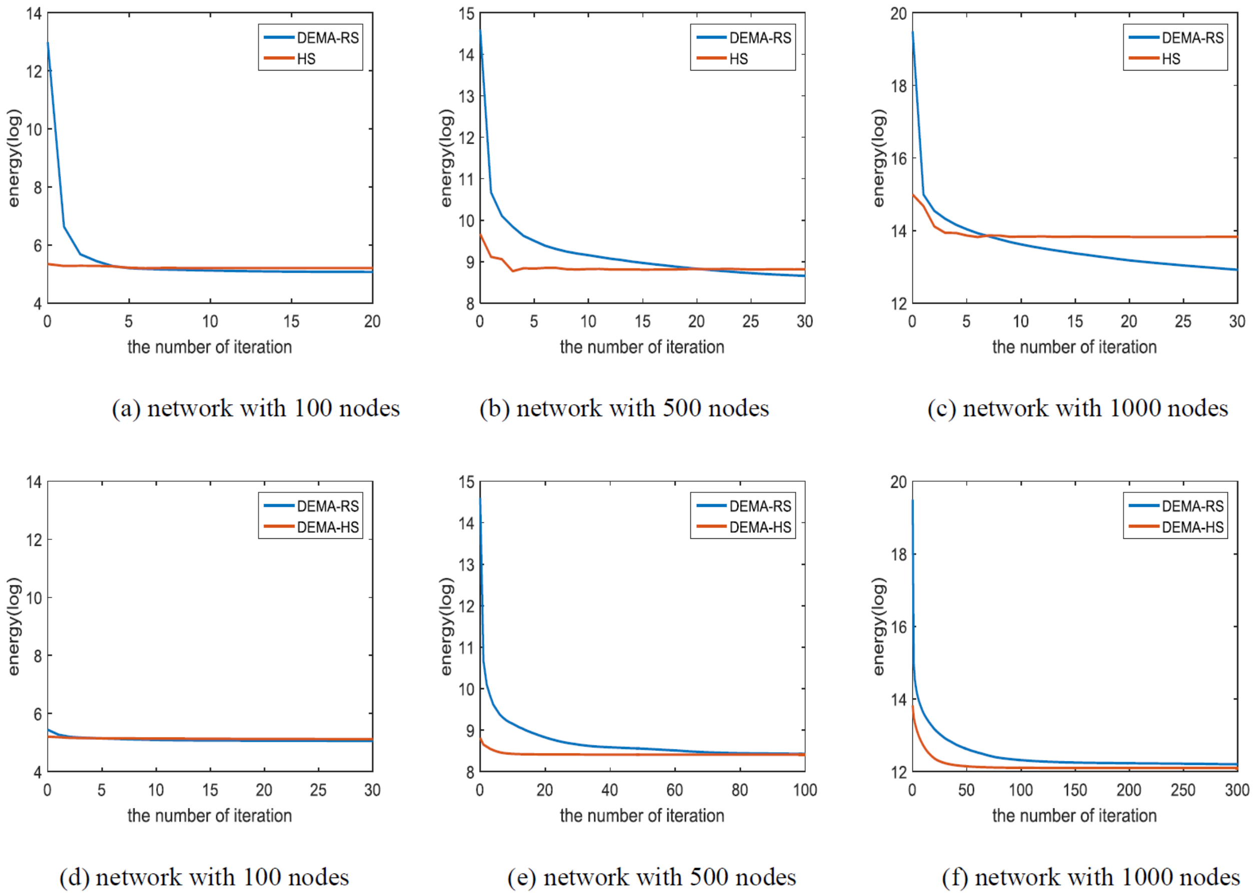

(a) network with 100 nodes (b) network with 500 nodes (c) network with 1000 nodes

(d) network with 100 nodes (e) network with 500 nodes (f) network with 1000 nodes

Fig_S4 Comparison of DEMA with HS and DEMA with RS. The heuristic start (HS) is compared with DEMA with random start (DEMA-RS). From (a)-(c), the results show that the HS can converge to a small energy within the 5 iteration when the energy of DEMA-RS is still high. However, after a few iterations, DEMA-RS can converge to a smaller energy. The layout generated by the HS can be used as an initial layout for DEMA. The layouts generated by the HS and DEMA-RS in the fourth iteration are both used as the initial layouts. (d)-(f) show that the DEMA-HS is faster than DEMA-RS to converge to a minimum.

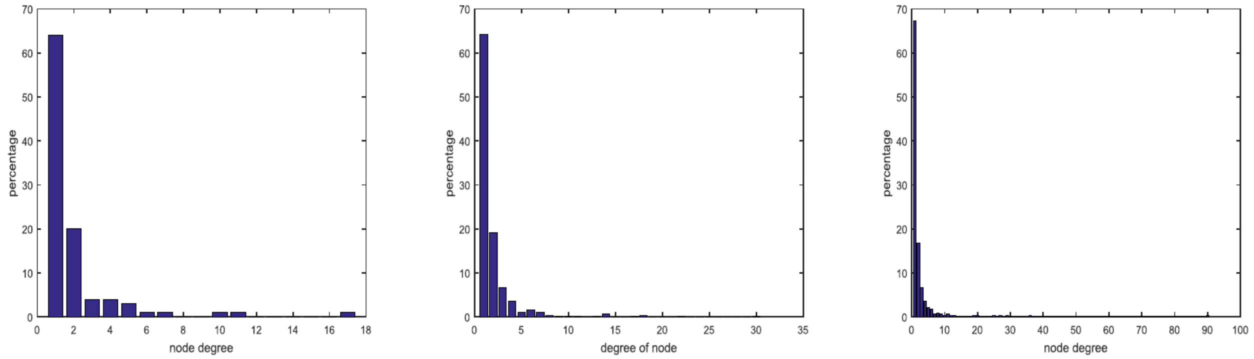

(a) 100 nodes (b) 500 nodes (a) 1000 nodes

Fig_S5 Distribution of the degrees of nodes. The percentage in the y-axis denotes the percentage of nodes with some degree against the total nodes. The distribution satisfies scale-free property.

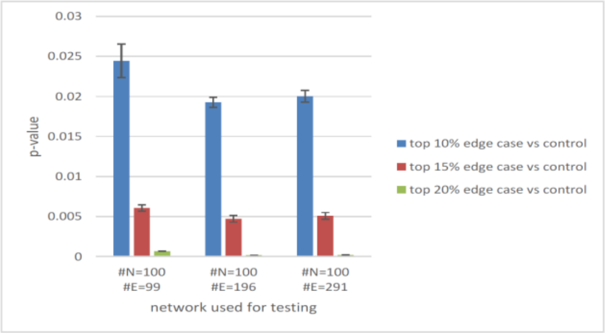

(a) 100 nodes

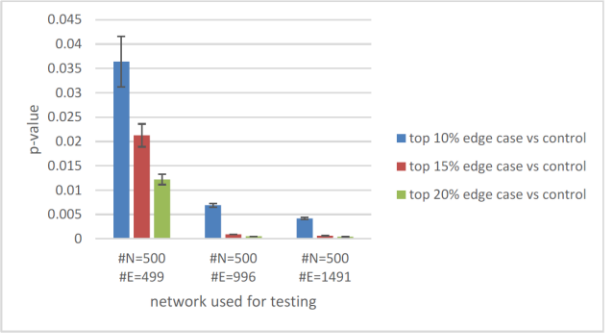

(b) 500 nodes

Fig_S6 Measures of the topological property. #N denotes the number of the nodes. #E denotes the number of the edges. (a) Space-filling (SF) is used to evaluate the visual clarity. The larger, the better. (b) Hub shrinking (HS) is used to evaluate the structural modularity. The smaller, the better. (c) and (d) use HS/SF to achieve a balance between the visual clarity and the structural modularity. The smaller, the better.

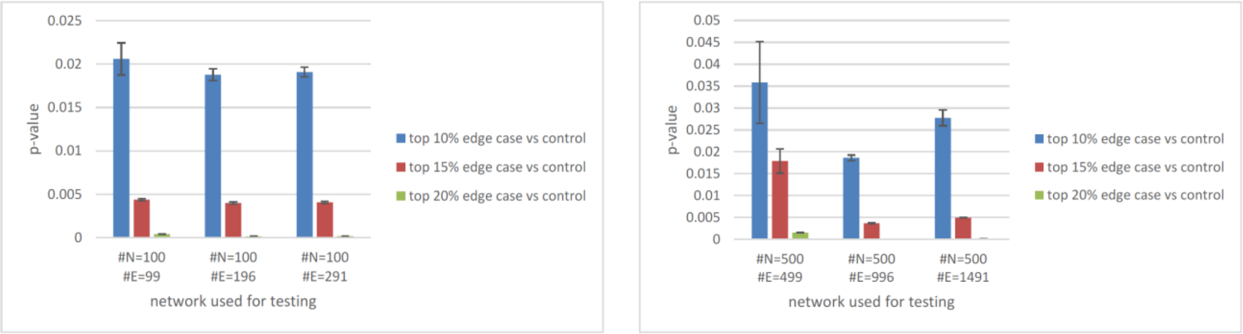

(a) 100 nodes (b) 500 nodes

Fig_S7 Measures of IC with different numbers of nodes. #N denotes the number of nodes. #E denotes the number of edges. (a) It is the case with 100 nodes with different numbers of edges. (b) It is the case with 500 nodes with different numbers of edges.

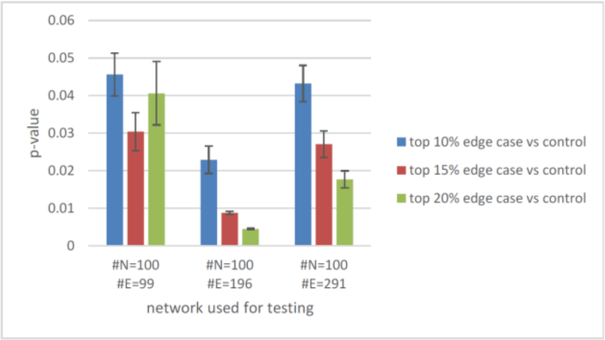

(a) 100 nodes

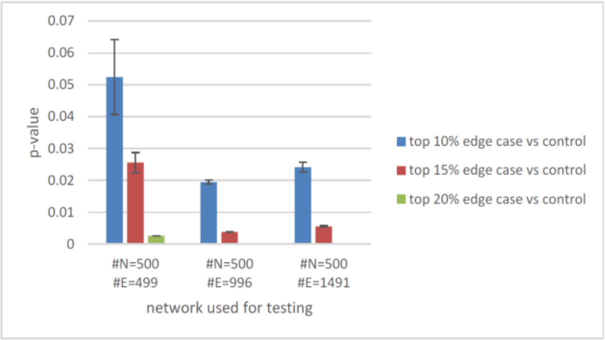

(b) 500 nodes

Fig_S8 Measures of EC with different numbers of nodes. #N denotes the number of nodes. #E denotes the number of edges. (a) It is the case with 100 nodes with different numbers of edges. (b) It is the case with 500 nodes with different numbers of edges.

Table. S1 The grouping performance comparison using inside mean value of squared distance (IMSD). SFARI represents the genes annotated by SFARI reference. De novo represents the genes annotated by de novo CNVs. They “OL” represents organic layout, “FD” represents force-directed layout, “EWFD” represents edge weighted force-directed layout, “FR” represents Fruchterman and Reingold algorithm and FA2 represents ForceAtlas2.

**Layout**	**IMSD**	
	**SFARI**	**de novo**
DEMA	**0.31**	**0.33**
OL	0.67	0.77
FD	0.66	0.79
EWFD	0.65	0.75
FA2	0.62	0.87
FR	0.99	0.92

Table. S2 The genes with over two shared PAGs from wiki-pathway highlighted in the DEMA layout of Alzheimer's disease. The "shared PAGs" consists of the PAG IDs delimited by "|".

Gene	Shared PAGs	Degree	Betweenness Centrality
TNF	WAG002021|WAG002004|WAG002729	83	4.58E-02
IL6	WAG002021|WAG002004|WAG002729	67	2.11E-02
ALB	WAG002021|WAG002786|WAG002004	51	2.83E-02
IL1B	WAG002021|WAG002004|WAG002729	48	1.52E-02
APOE	WAG002786|WAG002004|WAG002606|WAG002857	47	2.10E-02
APOA1	WAG002857|WAG002004|WAG002786|WAG002606|WAG002021	39	1.76E-02
ICAM1	WAG002021|WAG002004|WAG002729	39	1.42E-02
CCL2	WAG002021|WAG002004|WAG002729	37	5.22E-03
LPL	WAG002786|WAG002606|WAG002857	33	1.28E-02
APOB	WAG002857|WAG002004|WAG002786|WAG002606|WAG002021	31	8.24E-03
ABCA1	WAG002021|WAG002786|WAG002004|WAG002857	27	9.26E-03
APOA2	WAG002786|WAG002606|WAG002857	25	2.89E-03
APOC3	WAG002786|WAG002606|WAG002857	19	1.04E-03
LDLR	WAG002857|WAG002004|WAG002786|WAG002606|WAG002021	18	3.49E-03
SCARB1	WAG002021|WAG002004|WAG002857	14	7.65E-04
APOC2	WAG002786|WAG002606|WAG002857	11	1.79E-04
APOA4	WAG002786|WAG002606|WAG002857	6	0.00E+00
